# Concurrent Visual and Auditory Deficits in a Patient with *Rickettsia conorii* Infection

**DOI:** 10.4269/ajtmh.23-0173

**Published:** 2023-07-03

**Authors:** Uri Manor, Jonathan Kfir, Nir Livneh, Tal Zilberman, Dana Yelin, Eyal Meltzer

**Affiliations:** ^1^ Internal Medicine C, Sheba Medical Center, Tel HaShomer, Israel;; ^2^ Sackler Faculty of Medicine, Tel Aviv University, Tel Aviv, Israel;; ^3^ Goldschleger Eye Institute, Sheba Medical Center, Tel HaShomer, Israel;; ^4^ Department of Otolaryngology, Sheba Medical Center, Tel HaShomer, Israel;; ^5^ Infectious Disease Unit, Sheba Medical Center, Tel HaShomer, Israel

**Figure 1.**
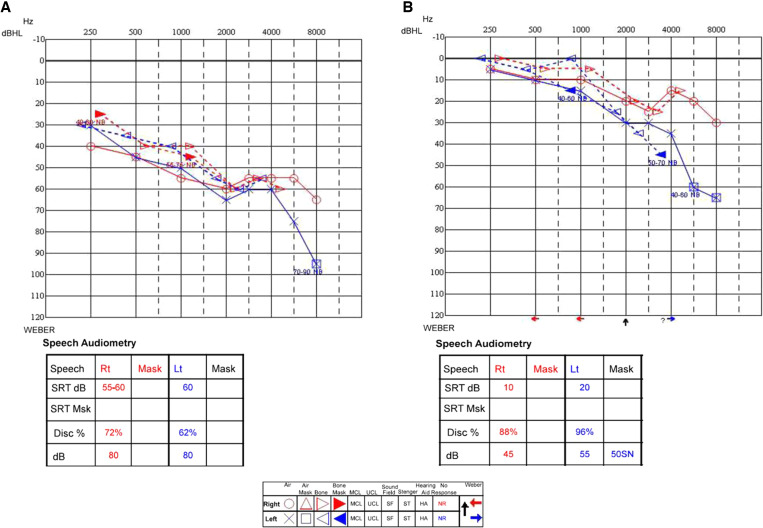
Audiometry. (**A**) Test done during active infection demonstrates a symmetric sensorineural hearing loss, sloping down from moderate in the lower tones to severe in the higher tones. Speech recognition tests show poor function with a threshold of 60 dB and word recognition score of 72% on the right ear and 62% on the left ear. (**B**) Same test that was done at the same center 4 months later shows much better results. The audiogram shows a pattern of presbycusis, slightly worse on the left ear, sloping down from normal to moderate, which probably represents the patient’s basic hearing function. Speech tests also improved with a threshold of up to 20 dB and word recognition score down to 88%, which is considered within the normal range. dB = decibels; dBHL = decibels hearing level; Lt = left; MCL = most comfortable loudness; NB = narrowband; SN = speech noise; SRT = speech recognition threshold; Rt = right; SRT Msk = speech recognition threshold masked; UCL = uncomfortable loudness.

**Figure 2.**
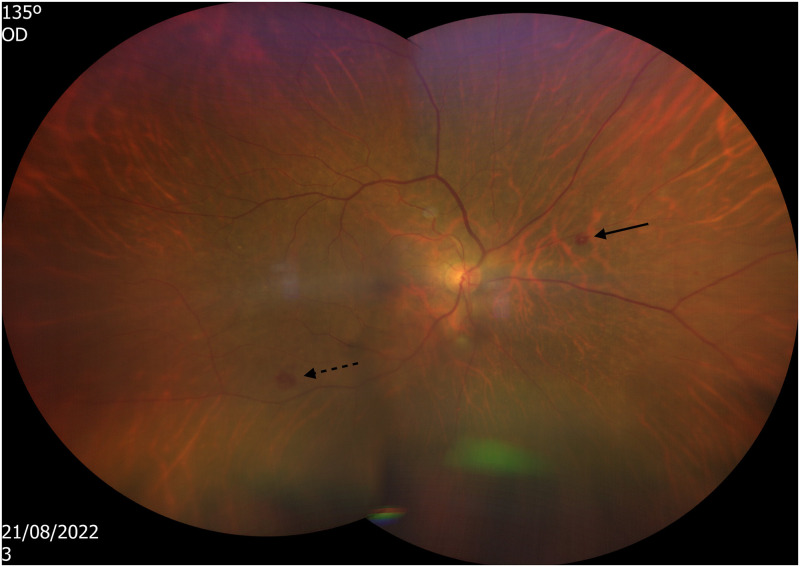
Fundoscopy. Right eye, white centered retinal hemorrhage (Roth’s spot) (arrow) and superficial hemorrhage (dashed arrow).

A 64-year-old woman presented with fever, shortness of breath, and gastrointestinal complaints. A laboratory workup revealed thrombocytopenia, elevated inflammatory markers, and liver enzyme abnormalities. While deteriorating on standard treatment of pneumonia, she developed a subtle maculopapular rash on her trunk and extremities, involving the palms and soles. Because a rickettsial infection was suspected, diagnostic tests were conducted, and treatment with doxycycline was initiated. A day later, she complained of new hearing loss and otodynia. A review of the treatment administered was devoid of known ototoxic medications, especially furosemide and aminoglycosides. Audiometry demonstrated a symmetric sensorineural hearing loss (Figure 1A). Assuming sensorineural hearing loss due to a rickettsial infection, we continued treatment with doxycycline alone, waving corticosteroids because of her septic condition. Positive serology for *Rickettsia conorii* (IgM +, IgG 1:400) and positive polymerase chain reaction for *R. conorii* from a skin biopsy verified our initial diagnosis, and she was discharged after improvement. Eleven days later, she presented again, now with a week-old complaint of blurry vision. On examination, the patient’s best corrected visual acuity (BCVA) was 20/50 in both eyes (BE; from a baseline of 20/20 BE 3 months prior). A slit lamp examination demonstrated unremarkable anterior chamber findings with no evidence of current or previous inflammation (BE). A dilated fundus examination revealed +2 vitreous cells BE and Roth’s spots in the right eye (Figure 2). She was readmitted, and treatment with doxycycline and prednisone (1 mg/kg) was initiated with marked clinical improvement. A subsequent ophthalmologic test 3 months later demonstrated only mild vitreous opacities BE, with a restored BCVA of 20/20. Audiometry 4 months later also showed striking improvement (Figure 1B).

Mediterranean spotted fever is an arthropod-borne disease caused by the obligate intracellular, gram-negative bacteria *R. conorii*. Classic manifestations of the disease include fever, maculopapular rash, and systemic symptoms. A severe presentation, with multiorgan failure and high mortality rates, is not uncommon.^1^ Central nervous system complications may occur via direct bacterial penetration or a vasculitic pathogenesis, leading to meningoencephalitis or sensorineural impairments.^2^ Central nervous system manifestations tend to occur more frequently in the elderly.^3^ Although rare, both auditory and visual impairments have been described after rickettsial infections.^4^^,^^5^ We attribute the auditory and visual improvement of our patient mainly to the corticosteroid treatment and time and less to the repeated doxycycline course, yet further research is needed to clarify this belief. To the best of our knowledge, our patient is the first to exhibit concurrent auditory and visual deficits secondary to a rickettsial infection.
